# *catena*-Poly[[[diaqua­(2-fluoro­benzoato-κ^2^ *O*,*O*′)strontium]-μ_3_-2-fluoro­benzoato-κ^5^ *O*:*O*,*O*′:*O*′,*F*] monohydrate]

**DOI:** 10.1107/S1600536811008397

**Published:** 2011-03-12

**Authors:** Zhu-Nian Jin

**Affiliations:** a College of Materials Science and Chemical Engineering, Jinhua College of Profession and Technology, Jinhua, Zhejiang 321017, People’s Republic of China

## Abstract

In the title compound, {[Sr(C_7_H_4_FO_2_)_2_(H_2_O)_2_]·H_2_O}_*n*_, the Sr^II^ atom is coordinated by six O atoms and one F atom from four 2-fluoro­benzoate ligands and two water mol­ecules, resulting in an irregular SrFO_8_ coordination environment. The μ_3_-2-fluoro­benzoate ligand bridges three symmetry-related Sr^II^ atoms, giving rise to a chain structure extending along [010]. The polymeric chains are connected *via* O—H⋯O hydrogen bonds into a two-dimensional supra­molecular structure parallel to (100).

## Related literature

For metal complexes with 2-fluoro­benzoate ligands, see: Zhang *et al.* (2005*a* ▶,*b* ▶); Zhang (2006 ▶). For related structures, see: Zhang (2008 ▶, 2009 ▶).
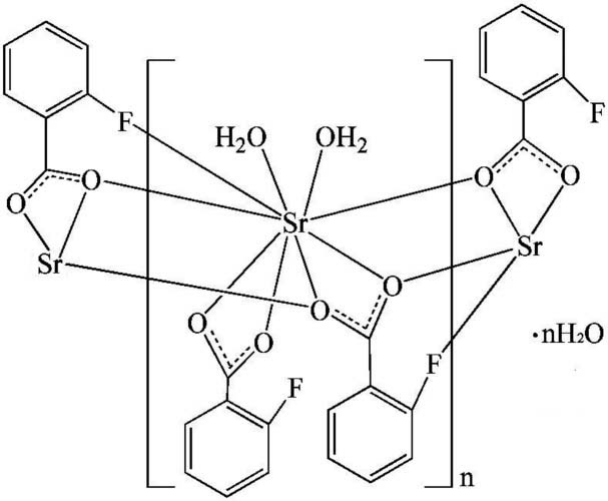


## Experimental

### 

#### Crystal data

[Sr(C_7_H_4_FO_2_)_2_(H_2_O)_2_]·H_2_O
*M* *_r_* = 419.87
Monoclinic, 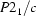

*a* = 12.515 (3) Å
*b* = 6.8232 (14) Å
*c* = 19.489 (4) Å
β = 93.71 (3)°
*V* = 1660.7 (6) Å^3^
*Z* = 4
Mo *K*α radiation
μ = 3.30 mm^−1^
*T* = 290 K
0.22 × 0.16 × 0.12 mm

#### Data collection

Rigaku R-AXIS RAPID diffractometer
Absorption correction: multi-scan (*ABSCOR*; Higashi, 1995 ▶) *T*_min_ = 0.535, *T*_max_ = 0.674
12414 measured reflections
2912 independent reflections
2351 reflections with *I* > 2σ(*I*)
*R*_int_ = 0.043

#### Refinement

*R*[*F* ^2^ > 2σ(*F* ^2^)] = 0.032
*wR*(*F* ^2^) = 0.097
*S* = 1.28
2912 reflections
218 parameters
H-atom parameters constrained
Δρ_max_ = 0.67 e Å^−3^
Δρ_min_ = −0.68 e Å^−3^

### 

Data collection: *RAPID-AUTO* (Rigaku, 1998 ▶); cell refinement: *RAPID-AUTO*; data reduction: *CrystalStructure* (Rigaku/MSC, 2002 ▶); program(s) used to solve structure: *SHELXS97* (Sheldrick, 2008 ▶); program(s) used to refine structure: *SHELXL97* (Sheldrick, 2008 ▶); molecular graphics: *SHELXTL* (Sheldrick, 2008 ▶); software used to prepare material for publication: *SHELXTL*.

## Supplementary Material

Crystal structure: contains datablocks I, global. DOI: 10.1107/S1600536811008397/hy2410sup1.cif

Structure factors: contains datablocks I. DOI: 10.1107/S1600536811008397/hy2410Isup2.hkl

Additional supplementary materials:  crystallographic information; 3D view; checkCIF report

## Figures and Tables

**Table 1:** Hydrogen-bond geometry (Å, °)

*D*—H⋯*A*	*D*—H	H⋯*A*	*D*⋯*A*	*D*—H⋯*A*
O5—H5*A*⋯O7^i^	0.82	2.11	2.854 (5)	152
O5—H5*B*⋯O7^ii^	0.82	2.14	2.914 (6)	158
O6—H6*A*⋯O1^iii^	0.82	2.10	2.791 (5)	142
O6—H6*B*⋯O2^ii^	0.82	2.22	2.901 (5)	140
O7—H7*A*⋯O2^ii^	0.82	2.01	2.800 (5)	161

Symmetry codes: (i) 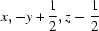; (ii) 
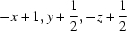
; (iii) 
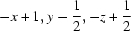
.

**Fig. 1.**
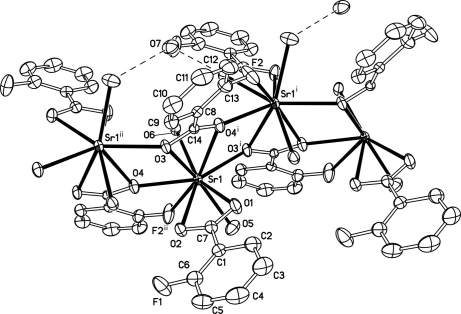
The one-dimensional chain structure of the title compound. Displacement ellipsoids are drawn at the 40% probability level. H atoms have been omitted for clarity. Hydrogen bonds are shown as dashed lines. [Symmetry codes: (i) -x+1, y+1/2, -z+1/2; (ii) -x+1, y-1/2, -z+1/2.]

**Fig. 2.**
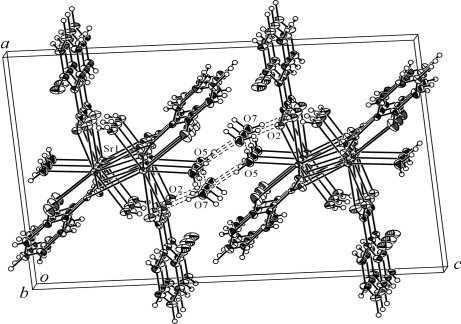
A packing diagram of the title compound, viewed along the b axis. Hydrogen bonds are shown as dashed lines.

## supplementary crystallographic information

### Comment

Metal ions with 2-fluorobenzoate ligands can form, among others, mononuclear and dinuclear complexes (Zhang, 2006; Zhang *et al.*, 2005*a*,b). However, very few complexes of 2-fluorobenzoate ligands with one-dimensional chain structure have been reported. In this paper, we report the synthesis and crystal structure of a one-dimensional chain strontium(II) complex with 2-fluorobenzoate.

The crystal structure of the title compound is similar to those of the reported complexes (Zhang, 2008, 2009). The Sr^II^ atom is coordinated by six O atoms and one F atom from four 2-fluorobenzoate ligands and two water molecules in an irregular SrFO_8_ coordination geometry. The µ_3_-2-fluorobenzoate ligand bridges three symmetry-related Sr^II^ atoms, giving rise to a chain structure extending along [0 1 0], with Sr—O bond lengths in the range of 2.463 (3) to 2.705 (3) Å and the Sr—F bond length being 2.908 (4) Å. Separation between Sr1 and Sr1^i^ [symmetry code: (i) -x+1, y+1/2, -z+1/2] is 4.1869 (8)Å (Fig. 1). The polymeric chains are connected via O—H···O intermolecular hydrogen bonds (Table 2) between the coordinated and uncoordinated water molecules and the carboxylate groups of the 2-fluorobenzoate ligands into a two-dimensional supramolecular structure (Fig. 2).

### Experimental

Sr(NO_3_)_2_ (0.109 g, 0.50 mmol) was dissolved in appropriate amount of water and then 1*M* Na_2_CO_3_ solution was added. SrCO_3_ was obtained by filtration, which was washed with distilled water for 5 times. The freshly prepared SrCO_3_, 2-fluorobenzoic acid (0.036 g, 0.25 mmol), 2,2'-bipyridine (0.039 g, 0.25 mmol) and CH_3_OH/H_2_O (v/v = 1:2, 15 ml) were mixed and stirred for 2 h. Subsequently, the resulting cream suspension was heated in a 23 ml Teflon-lined stainless steel autoclave at 433 K for 97 h. After the autoclave was cooled to room temperature in a procedure of 43 h, the solid was filtered off. The resulting filtrate was allowed to stand at room temperature, and slow evaporation for 6 weeks afforded colorless block single crystals.

### Refinement

C-bound H atoms were placed in calculated positions and refined as riding atoms, with C—H = 0.93 Å and *U*_iso_(H) = 1.2*U*_eq_(C). H atoms of water molecules were located in a difference Fourier map and refined with an O—H distance restraint of 0.82 (1) Å and with *U*_iso_(H) = 1.5*U*_eq_(O).

### Crystal data

**Table:**

[Sr(C_7_H_4_FO_2_)_2_(H_2_O)_2_]·H_2_O	*F*(000) = 840
*M**_r_* = 419.87	*D*_x_ = 1.679 Mg m^−^^3^
Monoclinic, *P*2_1_/*c*	Mo *K*α radiation, λ = 0.71073 Å
Hall symbol: -P 2ybc	Cell parameters from 2912 reflections
*a* = 12.515 (3) Å	θ = 3.2–25.0°
*b* = 6.8232 (14) Å	µ = 3.30 mm^−^^1^
*c* = 19.489 (4) Å	*T* = 290 K
β = 93.71 (3)°	Block, colorless
*V* = 1660.7 (6) Å^3^	0.22 × 0.16 × 0.12 mm
*Z* = 4	

### Data collection

**Table:**

Rigaku R-AXIS RAPID diffractometer	2912 independent reflections
Radiation source: rotation anode	2351 reflections with *I* > 2σ(*I*)
graphite	*R*_int_ = 0.043
ω scans	θ_max_ = 25.0°, θ_min_ = 3.2°
Absorption correction: multi-scan (*ABSCOR*; Higashi, 1995)	*h* = −14→14
*T*_min_ = 0.535, *T*_max_ = 0.674	*k* = −7→8
12414 measured reflections	*l* = −23→23

### Refinement

**Table:**

Refinement on *F*^2^	Secondary atom site location: difference Fourier map
Least-squares matrix: full	Hydrogen site location: inferred from neighbouring sites
*R*[*F*^2^ > 2σ(*F*^2^)] = 0.032	H-atom parameters constrained
*wR*(*F*^2^) = 0.097	*w* = 1/[σ^2^(*F*_o_^2^) + (0.*P*)^2^ + 5.2938*P*] where *P* = (*F*_o_^2^ + 2*F*_c_^2^)/3
*S* = 1.28	(Δ/σ)_max_ = 0.001
2912 reflections	Δρ_max_ = 0.67 e Å^−^^3^
218 parameters	Δρ_min_ = −0.68 e Å^−^^3^
0 restraints	Extinction correction: *SHELXL97* (Sheldrick, 2008), Fc^*^=kFc[1+0.001xFc^2^λ^3^/sin(2θ)]^-1/4^
Primary atom site location: structure-invariant direct methods	Extinction coefficient: 0.0066 (5)

### Fractional atomic coordinates and isotropic or equivalent isotropic displacement parameters (Å^2^)

**Table:**

	*x*	*y*	*z*	*U*_iso_*/*U*_eq_	
Sr1	0.49560 (4)	0.13650 (6)	0.18761 (2)	0.02804 (17)	
F1	0.8456 (3)	−0.2517 (6)	0.1198 (2)	0.0793 (13)	
F2	0.6409 (3)	0.4689 (5)	0.4237 (2)	0.0751 (12)	
O1	0.6953 (3)	0.2536 (5)	0.1848 (2)	0.0513 (11)	
O2	0.6718 (3)	−0.0557 (5)	0.15556 (19)	0.0398 (9)	
O3	0.5848 (3)	−0.0267 (4)	0.30415 (18)	0.0366 (8)	
O4	0.4430 (3)	−0.2113 (5)	0.18799 (19)	0.0386 (9)	
O5	0.5122 (3)	0.2790 (6)	0.0668 (2)	0.0581 (11)	
H5A	0.4729	0.2551	0.0325	0.087*	
H5B	0.5570	0.3656	0.0634	0.087*	
O6	0.3191 (3)	0.1157 (5)	0.24982 (19)	0.0414 (9)	
H6A	0.3064	0.0463	0.2827	0.062*	
H6B	0.3006	0.2265	0.2602	0.062*	
O7	0.3815 (3)	0.1569 (6)	0.4433 (2)	0.0602 (12)	
H7A	0.3644	0.2184	0.4082	0.090*	
H7B	0.3370	0.1511	0.4723	0.090*	
C1	0.8510 (4)	0.0722 (8)	0.1647 (3)	0.0356 (12)	
C2	0.9161 (5)	0.2282 (10)	0.1858 (3)	0.0525 (16)	
H2	0.8851	0.3423	0.2014	0.063*	
C3	1.0267 (5)	0.2175 (12)	0.1841 (4)	0.069 (2)	
H3	1.0689	0.3235	0.1989	0.082*	
C4	1.0738 (5)	0.0520 (13)	0.1607 (4)	0.069 (2)	
H4	1.1478	0.0459	0.1593	0.083*	
C5	1.0123 (4)	−0.1044 (11)	0.1394 (3)	0.0595 (18)	
H5	1.0439	−0.2176	0.1235	0.071*	
C6	0.9030 (4)	−0.0920 (9)	0.1419 (3)	0.0472 (14)	
C7	0.7313 (4)	0.0909 (7)	0.1680 (2)	0.0340 (12)	
C8	0.6861 (4)	0.1429 (7)	0.3916 (2)	0.0301 (11)	
C9	0.7526 (4)	−0.0162 (8)	0.4058 (3)	0.0406 (13)	
H9	0.7459	−0.1266	0.3779	0.049*	
C10	0.8289 (5)	−0.0148 (10)	0.4606 (3)	0.0572 (17)	
H10	0.8724	−0.1236	0.4694	0.069*	
C11	0.8399 (5)	0.1477 (12)	0.5016 (3)	0.0640 (19)	
H11	0.8915	0.1485	0.5382	0.077*	
C12	0.7760 (5)	0.3100 (10)	0.4897 (3)	0.0594 (18)	
H12	0.7830	0.4197	0.5180	0.071*	
C13	0.7012 (4)	0.3044 (8)	0.4344 (3)	0.0429 (13)	
C14	0.6027 (3)	0.1360 (7)	0.3328 (2)	0.0241 (10)	

### Atomic displacement parameters (Å^2^)

**Table:**

	*U*^11^	*U*^22^	*U*^33^	*U*^12^	*U*^13^	*U*^23^
Sr1	0.0313 (3)	0.0191 (2)	0.0333 (3)	−0.00040 (19)	−0.00080 (18)	0.0008 (2)
F1	0.050 (2)	0.060 (2)	0.128 (4)	0.0046 (19)	0.008 (2)	−0.038 (2)
F2	0.105 (3)	0.046 (2)	0.069 (3)	0.020 (2)	−0.030 (2)	−0.0262 (19)
O1	0.043 (2)	0.030 (2)	0.082 (3)	−0.0060 (18)	0.011 (2)	−0.015 (2)
O2	0.037 (2)	0.0299 (19)	0.052 (2)	−0.0019 (16)	0.0028 (17)	−0.0079 (17)
O3	0.049 (2)	0.0202 (17)	0.039 (2)	−0.0012 (16)	−0.0090 (17)	−0.0037 (15)
O4	0.044 (2)	0.0245 (18)	0.045 (2)	−0.0049 (16)	−0.0090 (17)	−0.0026 (16)
O5	0.063 (3)	0.068 (3)	0.043 (2)	−0.017 (2)	−0.001 (2)	0.013 (2)
O6	0.0369 (19)	0.0283 (19)	0.060 (2)	−0.0018 (16)	0.0105 (17)	0.0053 (18)
O7	0.072 (3)	0.064 (3)	0.045 (2)	0.014 (2)	0.004 (2)	0.013 (2)
C1	0.030 (3)	0.043 (3)	0.033 (3)	−0.002 (2)	0.000 (2)	0.001 (2)
C2	0.044 (3)	0.062 (4)	0.051 (4)	−0.012 (3)	0.001 (3)	−0.012 (3)
C3	0.040 (4)	0.097 (6)	0.069 (5)	−0.027 (4)	0.000 (3)	−0.012 (4)
C4	0.028 (3)	0.112 (6)	0.068 (5)	−0.001 (4)	0.005 (3)	−0.003 (4)
C5	0.032 (3)	0.085 (5)	0.061 (4)	0.016 (3)	0.007 (3)	−0.007 (4)
C6	0.036 (3)	0.057 (4)	0.048 (3)	−0.007 (3)	−0.001 (3)	−0.006 (3)
C7	0.039 (3)	0.035 (3)	0.026 (3)	−0.003 (2)	−0.009 (2)	−0.001 (2)
C8	0.032 (3)	0.029 (2)	0.030 (3)	−0.003 (2)	0.007 (2)	0.002 (2)
C9	0.038 (3)	0.041 (3)	0.042 (3)	0.006 (2)	−0.004 (2)	0.005 (3)
C10	0.042 (3)	0.073 (5)	0.055 (4)	0.016 (3)	−0.006 (3)	0.013 (4)
C11	0.049 (4)	0.098 (6)	0.043 (4)	−0.005 (4)	−0.011 (3)	0.000 (4)
C12	0.069 (4)	0.071 (5)	0.037 (4)	−0.005 (4)	−0.009 (3)	−0.012 (3)
C13	0.044 (3)	0.042 (3)	0.041 (3)	0.002 (3)	−0.003 (3)	−0.006 (3)
C14	0.021 (2)	0.023 (2)	0.029 (2)	0.000 (2)	0.0043 (18)	0.000 (2)

### Geometric parameters (Å, °)

**Table:**

Sr1—O4	2.463 (3)	C1—C6	1.384 (8)
Sr1—O3^i^	2.518 (3)	C1—C2	1.387 (7)
Sr1—O5	2.568 (4)	C1—C7	1.509 (7)
Sr1—O6	2.591 (3)	C2—C3	1.388 (8)
Sr1—O1	2.627 (4)	C2—H2	0.9300
Sr1—O2	2.674 (3)	C3—C4	1.365 (10)
Sr1—O4^i^	2.703 (4)	C3—H3	0.9300
Sr1—O3	2.705 (3)	C4—C5	1.364 (9)
Sr1—F2^ii^	2.908 (4)	C4—H4	0.9300
F1—C6	1.359 (6)	C5—C6	1.375 (7)
F2—C13	1.361 (6)	C5—H5	0.9300
F2—Sr1^i^	2.908 (4)	C8—C9	1.385 (7)
O1—C7	1.250 (6)	C8—C13	1.387 (7)
O2—C7	1.261 (6)	C8—C14	1.500 (6)
O3—C14	1.256 (5)	C9—C10	1.386 (8)
O3—Sr1^ii^	2.518 (3)	C9—H9	0.9300
O4—C14^ii^	1.244 (5)	C10—C11	1.369 (9)
O4—Sr1^ii^	2.703 (4)	C10—H10	0.9300
O5—H5A	0.8200	C11—C12	1.377 (9)
O5—H5B	0.8200	C11—H11	0.9300
O6—H6A	0.8200	C12—C13	1.382 (8)
O6—H6B	0.8200	C12—H12	0.9300
O7—H7A	0.8200	C14—O4^i^	1.244 (5)
O7—H7B	0.8200		
			
O4—Sr1—O3^i^	140.48 (11)	H6A—O6—H6B	105.5
O4—Sr1—O5	113.89 (13)	H7A—O7—H7B	116.6
O3^i^—Sr1—O5	76.65 (13)	C6—C1—C2	115.9 (5)
O4—Sr1—O6	73.14 (11)	C6—C1—C7	124.8 (5)
O3^i^—Sr1—O6	70.37 (11)	C2—C1—C7	119.3 (5)
O5—Sr1—O6	124.86 (13)	C1—C2—C3	121.4 (6)
O4—Sr1—O1	123.21 (12)	C1—C2—H2	119.3
O3^i^—Sr1—O1	96.20 (11)	C3—C2—H2	119.3
O5—Sr1—O1	74.49 (14)	C4—C3—C2	120.2 (6)
O6—Sr1—O1	150.09 (12)	C4—C3—H3	119.9
O4—Sr1—O2	75.68 (11)	C2—C3—H3	119.9
O3^i^—Sr1—O2	143.25 (11)	C5—C4—C3	120.1 (6)
O5—Sr1—O2	81.65 (13)	C5—C4—H4	119.9
O6—Sr1—O2	145.41 (11)	C3—C4—H4	119.9
O1—Sr1—O2	49.04 (11)	C4—C5—C6	119.0 (6)
O4—Sr1—O4^i^	115.34 (9)	C4—C5—H5	120.5
O3^i^—Sr1—O4^i^	71.56 (10)	C6—C5—H5	120.5
O5—Sr1—O4^i^	129.93 (12)	F1—C6—C5	116.5 (6)
O6—Sr1—O4^i^	78.62 (11)	F1—C6—C1	120.0 (5)
O1—Sr1—O4^i^	71.71 (12)	C5—C6—C1	123.4 (6)
O2—Sr1—O4^i^	101.95 (11)	O1—C7—O2	122.4 (5)
O4—Sr1—O3	72.34 (10)	O1—C7—C1	117.5 (4)
O3^i^—Sr1—O3	117.84 (8)	O2—C7—C1	120.0 (5)
O5—Sr1—O3	150.73 (12)	O1—C7—Sr1	60.2 (3)
O6—Sr1—O3	84.40 (11)	O2—C7—Sr1	62.3 (3)
O1—Sr1—O3	78.54 (12)	C1—C7—Sr1	175.0 (3)
O2—Sr1—O3	72.10 (11)	C9—C8—C13	116.5 (5)
O4^i^—Sr1—O3	47.70 (10)	C9—C8—C14	120.5 (4)
O4—Sr1—F2^ii^	58.61 (10)	C13—C8—C14	123.1 (4)
O3^i^—Sr1—F2^ii^	100.89 (11)	C8—C9—C10	121.6 (6)
O5—Sr1—F2^ii^	62.81 (12)	C8—C9—H9	119.2
O6—Sr1—F2^ii^	81.40 (12)	C10—C9—H9	119.2
O1—Sr1—F2^ii^	128.15 (13)	C11—C10—C9	119.6 (6)
O2—Sr1—F2^ii^	94.95 (12)	C11—C10—H10	120.2
O4^i^—Sr1—F2^ii^	160.00 (12)	C9—C10—H10	120.2
O3—Sr1—F2^ii^	130.94 (9)	C10—C11—C12	121.1 (6)
C13—F2—Sr1^i^	137.0 (3)	C10—C11—H11	119.4
C7—O1—Sr1	95.5 (3)	C12—C11—H11	119.4
C7—O2—Sr1	93.0 (3)	C11—C12—C13	117.8 (6)
C14—O3—Sr1^ii^	147.1 (3)	C11—C12—H12	121.1
C14—O3—Sr1	93.5 (3)	C13—C12—H12	121.1
Sr1^ii^—O3—Sr1	106.53 (12)	F2—C13—C12	116.0 (5)
C14^ii^—O4—Sr1	157.5 (3)	F2—C13—C8	120.7 (5)
C14^ii^—O4—Sr1^ii^	93.9 (3)	C12—C13—C8	123.3 (5)
Sr1—O4—Sr1^ii^	108.21 (12)	O4^i^—C14—O3	122.0 (4)
Sr1—O5—H5A	126.3	O4^i^—C14—C8	120.3 (4)
Sr1—O5—H5B	116.3	O3—C14—C8	117.6 (4)
H5A—O5—H5B	117.0	O4^i^—C14—Sr1	62.1 (2)
Sr1—O6—H6A	127.7	O3—C14—Sr1	62.2 (2)
Sr1—O6—H6B	109.2	C8—C14—Sr1	162.0 (3)

Symmetry codes: (i) −*x*+1, *y*+1/2, −*z*+1/2; (ii) −*x*+1, *y*−1/2, −*z*+1/2.

### Hydrogen-bond geometry (Å, °)

**Table:**

*D*—H···*A*	*D*—H	H···*A*	*D*···*A*	*D*—H···*A*
O5—H5A···O7^iii^	0.82	2.11	2.854 (5)	152
O5—H5B···O7^i^	0.82	2.14	2.914 (6)	158
O6—H6A···O1^ii^	0.82	2.10	2.791 (5)	142
O6—H6B···O2^i^	0.82	2.22	2.901 (5)	140
O7—H7A···O2^i^	0.82	2.01	2.800 (5)	161

Symmetry codes: (iii) *x*, −*y*+1/2, *z*−1/2; (i) −*x*+1, *y*+1/2, −*z*+1/2; (ii) −*x*+1, *y*−1/2, −*z*+1/2.
